# Comparison of one-week versus three-week paclitaxel for advanced pan-carcinomas: systematic review and meta-analysis

**DOI:** 10.18632/aging.203919

**Published:** 2022-02-26

**Authors:** Shitong Lin, Ting Peng, Yifan Meng, Canhui Cao, Peipei Gao, Ping Wu, Wenhua Zhi, Ye Wei, Tian Chu, Binghan Liu, Juncheng Wei, Xiaoyuan Huang, Wencheng Ding, Cai Cheng

**Affiliations:** 1 Cancer Biology Research Center (Key Laboratory of the Ministry of Education), Tongji Hospital, Tongji Medical College, Huazhong University of Science and Technology, Wuhan, Hubei, China; 2 Department of Gynecologic Oncology, Tongji Hospital, Tongji Medical College, Huazhong University of Science and Technology, Wuhan, Hubei, China; 3 Department of Gynecologic Oncology, State Key Laboratory of Oncology in South China, Collaborative Innovation Center for Cancer Medicine, Sun Yat-Sen University Cancer Center, Guangzhou, China; 4 Department of Reproductive Medicine, Peking University Shenzhen Hospital, Shenzhen, Guangdong Province, China; 5 Division of Cardiothoracic and Vascular Surgery, Tongji Hospital, Tongji Medical College, Huazhong University of Science and Technology, Wuhan, Hubei, China

**Keywords:** paclitaxel, one-week, three-week, pan-carcinomas, meta-analysis

## Abstract

Paclitaxel remains the first-line chemotherapy regimen for many malignant tumors. However, prognosis and adverse events under different dosing regimens (one-week versus three-week treatment) remain contradictory in many randomized controlled trials (RCTs). Here, we performed a comprehensive meta-analysis to measure the efficacy and toxicities of these two dosing regimens. Four databases were systematically retrieved. RCTs comparing two paclitaxel dosing regimens for advanced malignant tumors with assessable outcomes (e.g., overall survival (OS), progression-free survival (PFS), toxicities, response rates) were included. In total, 19 eligible RCTs involving 9 674 patients were included. Meta-analysis of pan-cancers revealed that weekly paclitaxel treatment was more beneficial regarding PFS compared to three-week paclitaxel treatment (hazard ratio (HR) = 0.90, 95% confidence interval (CI) = 0.82–0.99, *P* = 0.02). Nevertheless, there was no significant difference in terms of OS between the two dosing regimens (HR = 0.98, 95%CI = 0.91–1.06, *P* = 0.62) or other tested subgroups. In terms of serious adverse events, grade 3 or 4 (G3/4) neutropenia, G3/4 febrile neutropenia, G3/4 arthritis, and G3/4 alopecia occurred less often under weekly paclitaxel treatment. In summary, Weekly paclitaxel treatment demonstrates better PFS and fewer chemotherapy-induced hematological and non-hematological toxicities compared to the three-week paclitaxel regimen.

## INTRODUCTION

Malignant tumors remain one of the major causes of shortened human life expectancy. As of 2020, it is estimated there will be 19.3 million new cancer cases and 10.0 million new cancer deaths worldwide each year [1]. Paclitaxel is a first-line chemotherapy drug commonly used to control progression of advanced malignancies (e.g., ovarian cancer, breast cancer, cervical cancer, endometrial cancer, and non-small cell lung carcinoma), with a standard dosing schedule of once every three weeks [2–6]. Recently, several randomized controlled trials (RCTs) have indicated that a weekly paclitaxel regimen could significantly improve patient prognosis or decrease serious adverse events [7–9]. The latest edition of the National Comprehensive Cancer Network (NCCN) guidelines also recommend weekly paclitaxel for HER2-negative breast cancer and ovarian cancer [10–12]. However, other RCTs have found no significant differences between the two paclitaxel regimens in regard to prognosis and adverse events [13, 14]. Thus, RCT and meta-analysis outcomes are not entirely consistent, and clinicians do not have clear information as to the most appropriate paclitaxel administration schedule. Identifying the optimal paclitaxel administration schedule is critical for patient care and survival.

A meta-analysis of pan-carcinomas comparing the overall response rate (ORR) and toxicities between one-week and three-week paclitaxel regimens was published in 2012 [15]. However, the conclusions reached in that research mistakenly suggested that weekly paclitaxel favored a better ORR due to an error in the forest plot. Thus, in the current study, we performed a systematic meta-analysis to measure the appropriate dose and schedule regarding prognosis (e.g., overall survival (OS) and progression-free survival (PFS) and serious adverse events.

## RESULTS

**Figure 1:**
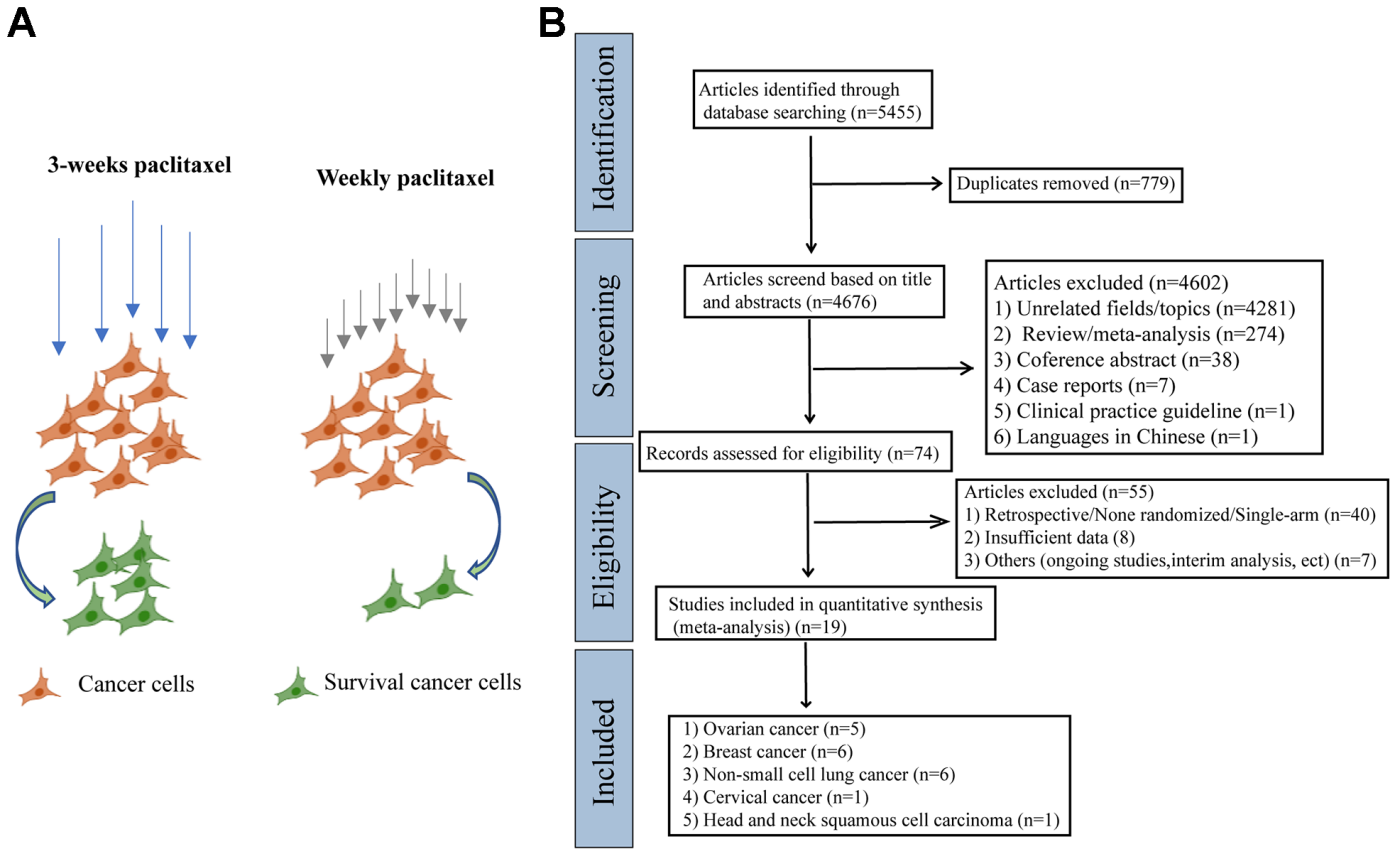
**The schematic diagram of potential mechanism of different paclitaxel regimens and PRISMA flow diagram.** (**A**) The schematic diagram of the effects of different paclitaxel administration schedules on the survival of cancer cells. (**B**) Flowchart of the literature search for the 19 eligible RCTs comparing therapeutic efficacy of weekly paclitaxel and 3-weeks paclitaxel administration schedules.

At the beginning of the meta-analysis, we consulted a large number of literatures and found that weekly paclitaxel may be more effective in killing tumor cells compared to 3-weeks paclitaxel [16–18]. We therefore made a schematic diagram showing the difference of different paclitaxel regimens in killing cancer cells (Figure 1A). As shown in Figure 1B, 5 455 articles were identified in the four databases (Cochrane Library, PubMed, Web of Science, and Clinical Trials.gov). In total, 19 RCTs containing 9 674 patients were included in our meta-analysis after excluding ineligible RCTs [7–9, 13, 14, 19–32].

### Characteristics of included RCTs

As shown in Table 1, our meta-analysis included five ovarian cancer RCTs, six breast cancer RCTs, six non-small cell lung cancer RCTs, one cervical cancer RCT, and one head and neck squamous cell carcinoma RCT. Among these RCTs, 11 were phase III RCTs, three were phase II RCTs, and five RCTs did not clearly indicate in which phase they belonged. These eligible RCTs were mainly carried out in America, India, China, and Japan, and primarily involved Asian and European races. The number of enrolled patients varied greatly among RCTs, ranging from 46 to 2 484. One cervical cancer RCT presented in the form of a conference abstract only contained data on the response rate [28]. Overall, 5 059 and 4 615 patients were assigned to the one-week and three-week paclitaxel regimens, respectively.

**Table 1:** The characteristics of eligible clinical trials.

**Study**	**Country**	**Phase**	**Cancer**	**Group**	**No.**	**Paclitaxel schedules**	**Combined anti-cancer drug and dosage regime**
Rosenberg 2002	Sweden	II	Ovarian cancer	Weekly	105	67 mg/m^2^[d1,8,15 q3w]	No
				3-weeks	103	200 mg/m^2^[d1 q3w]	
Katsumata 2013	Japan	III	Ovarian cancer	Weekly	312	80 mg/m^2^[d1,8,15 q3w]	Carboplatin AUC 6 mg/mL per min [d1 q3w]
				3-weeks	319	175 mg/m^2^[d1 q3w]	Carboplatin AUC 6 mg/mL per min [d1 q3w]
Pignata 2014	Italy and France	III	Ovarian cancer	Weekly	406	60 mg/m^2^[d1,8,15 q3w]	Carboplatin AUC 2 mg/mL per min [d1,8,15 q3w]
				3-weeks	404	175 mg/m^2^[d1 q3w]	Carboplatin AUC 6 mg/mL per min [d1 q3w]
Chan 2016	America	III	Ovarian cancer	Weekly	346	80 mg/m^2^[d1,8,15 q3w]	Carboplatin AUC 6 mg/mL per min [d1 q3w]
				3-weeks	346	175 mg/m^2^[d1 q3w]	Carboplatin AUC 6 mg/mL per min [d1 q3w]
Clamp 2019	Britain	III	Ovarian cancer	Weekly	522	80 mg/m^2^[d1,8,15 q3w]	Carboplatin AUC 5 or 6 mg/mL per min [d1 q3w]
				Weekly	521	80 mg/m^2^[d1,8,15 q3w]	Carboplatin AUC 2 mg/mL per min [d1 q3w]
				3-weeks	522	175 mg/m^2^[d1 q3w]	Carboplatin AUC 5 or 6 mg/mL per min [d1 q3w]
Green 2005	America	NA	Breast cancer	Weekly	127	80 mg/m^2^[d1,8,15 q3w] or 150 mg/m^2^[d1,8,15 q4w]	Fluorouracil 500 mg/m^2^& Cyclophosphamide 500 mg/m^2^& Doxorubicin 50 mg/m^2^[d1 q3w]
				3-weeks	131	225 mg/m^2^[d1 q3w]	Fluorouracil 500 mg/m^2^& Cyclophosphamide 500 mg/m^2^& Doxorubicin 50 mg/m^2^[d1 q3w]
Perez 2005	America	II	Breast cancer	Weekly	48	80 mg/m^2^[d1,8,15 q4w]	Carboplatin 2 mg/mL per min [d1,8,15 q4w] & Trastuzumab 2-4 mg/m^2^[d1,8,15,22 q4w]
				3-weeks	43	200 mg/m^2^[d1 q3w]	Carboplatin 6 mg/mL per min & Trastuzumab 6-8 mg/m^2^[d1 q3w]
Seidman 2008	America	III	Breast cancer	Weekly	346	80 mg/m^2^[d1,8,15 q3w]	Trastuzumab (patients were stratified by HER-2-status for trastuzumab schedule)
				3-weeks	383	175 mg/m^2^[d1 q3w]	
Sparano 2008	America	NA	Breast cancer	Weekly	1231	80 mg/m^2^[q1w for 12 doses]	Doxorubicin 60 mg/m^2^& Cyclophosphamide 600 mg/m^2^[d1 q3w]
				3-weeks	1253	175 mg/m^2^[q3w for 4 doses]	Doxorubicin 60 mg/m^2^& Cyclophosphamide 600 mg/m^2^[d1 q3w]
Qi 2010	China	NA	Breast cancer	Weekly	104	60 mg/m^2^[d1,8,15 q3w]	Carboplatin AUC 6 mg/mL per min [d1 q3w]
				3-weeks	109	175 mg/m^2^[d1 q3w]	Carboplatin AUC 6 mg/mL per min [d1 q3w]
Yu 2013	China	NA	Breast cancer	Weekly	29	80 mg/m^2^[d1,8,15 q4w]	Carboplatin AUC 2 mg/mL per min & Trastuzumab 2mg/kg [d1,8,15 q4w]
				3-weeks	27	175 mg/m^2^[d1 q3w]	Carboplatin AUC 6 mg/mL per min & Trastuzumab 6mg/kg [d1 q3w]
Schuette 2006	Germany	III	NSCLC	Weekly	434	100 mg/m^2^[weekly for 6-8 weeks]	Carboplatin AUC 2 mg/mL per min [weekly for 6-8 weeks]
				3-weeks	449	200 mg/m^2^[d1 q3w]	Carboplatin AUC 6 mg/mL per min [d1 q3w]
Socinski 2006	America	III	NSCLC	Weekly	80	75 mg/m^2^[weekly*12 cycles]	Carboplatin AUC 6 mg/mL per min [d1 q3w * 4 cycles]
				3-weeks	81	225 mg/m^2^[q3w*4cycles]	Carboplatin AUC 6 mg/mL per min [d1 q3w * 4 cycles]
Belani 2007	America	III	NSCLC	Weekly	51	100 mg/m^2^[d1,8 q3w]	Gemcitabine 1000 mg/m^2^ [d1,8 q3w]
				3-weeks	52	200 mg/m^2^[d1 q3w]	Gemcitabine 1000 mg/m^2^ [d1,8 q3w]
Belani 2008	America	III	NSCLC	Weekly	223	100 mg/m^2^[d1,8,15 q4w]	Carboplatin AUC 6 mg/mL per min [d1 q3w]
				3-weeks	221	225 mg/m^2^[d1 q3w]	Carboplatin AUC 6 mg/mL per min [d1 q3w]
Socinski 2009	America	III	NSCLC	Weekly	84	100 mg/m^2^[d1,8,15 q4w]	Carboplatin AUC 6 mg/mL per min [d1 q3w]; Cetuximab [400 mg/m^2^ day 1 followed by weekly 250 mg/m^2^]
				3-weeks	84	225 mg/m^2^[d1 q3w]	Carboplatin AUC 6 mg/mL per min [d1 q3w]; Cetuximab [400 mg/m^2^ day 1 followed by weekly 250 mg/m^2^]
Sakakibara 2010	Japan	III	NSCLC	Weekly	42	70 mg/m^2^[d1,8,15 q4w]	Carboplatin AUC 6 mg/mL per min [d1 q3w]
				3-weeks	40	200 mg/m^2^[d1 q3w]	Carboplatin AUC 6 mg/mL per min [d1 q3w]
Mathur 2018	India	NA	HNSCC	Weekly	25	80 mg/m^2^[d1,8,15 q3w]	Carboplatin AUC 5 mg/mL per min [d1 q3w]
				3-weeks	25	175 mg/m^2^[d1 q3w]	Carboplatin AUC 5 mg/mL per min [d1 q3w]
Pathalingappa 2015	India	II	Cervical Cancer	Weekly	23	60 mg/m^2^[d1,8,15 q3w]	Carboplatin AUC 2 mg/mL per min [d1,8,15 q3w]
				3-weeks	23	175 mg/m^2^[d1 q3w]	Carboplatin AUC 6 mg/mL per min [d1 q3w]

NSCLC, Non-small cell lung cancer; HNSCC, Head and neck squamous cell carcinoma; NA, not available.

Paclitaxel and carboplatin were the main adjuvant chemotherapy drugs in the RCTs. Cetuximab, trastuzumab, doxorubicin, and cyclophosphamide were also used as combination drugs in some RCTs. There were some differences in the one-week (e.g., 60, 67, 70, 75, 80, 100, and 150 mg/m^2^) and three-week paclitaxel regimen doses (e.g., 175, 200, and 225 mg/m^2^). The doses of carboplatin also varied greatly in the different RCTs. The primary outcomes of the various RCTs also differed. Ten trials displayed data efficacy (OS or PFS), toxic events, and response rates in the original manuscript, and the six non-small cell lung cancer RCTs measured all these outcomes. Two breast cancer RCTs from China by Qi et al. and Yu et al. only included PRR and toxicity data, respectively [13, 23]. Furthermore, only PRR could be measured in the head and neck squamous cell carcinoma and cervical cancer RCTs.

### Comparative effectiveness of one-week versus three-week paclitaxel treatment weekly paclitaxel favored better PFS

**Figure 2:**
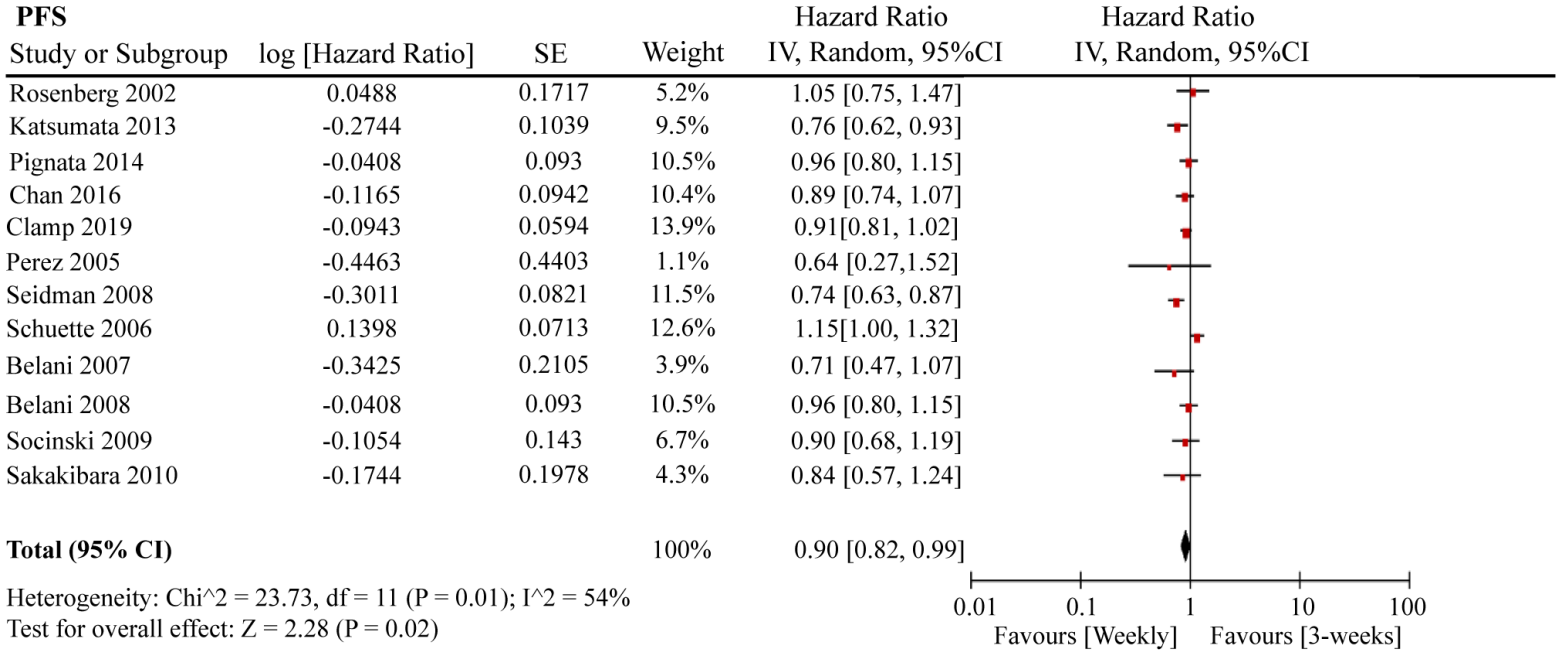
**The forest plot of HR for PFS in the weekly paclitaxel compared to 3-weeks paclitaxel regimen.** HR: hazard ratio; PFS: progression-free survival.

**Figure 3:**
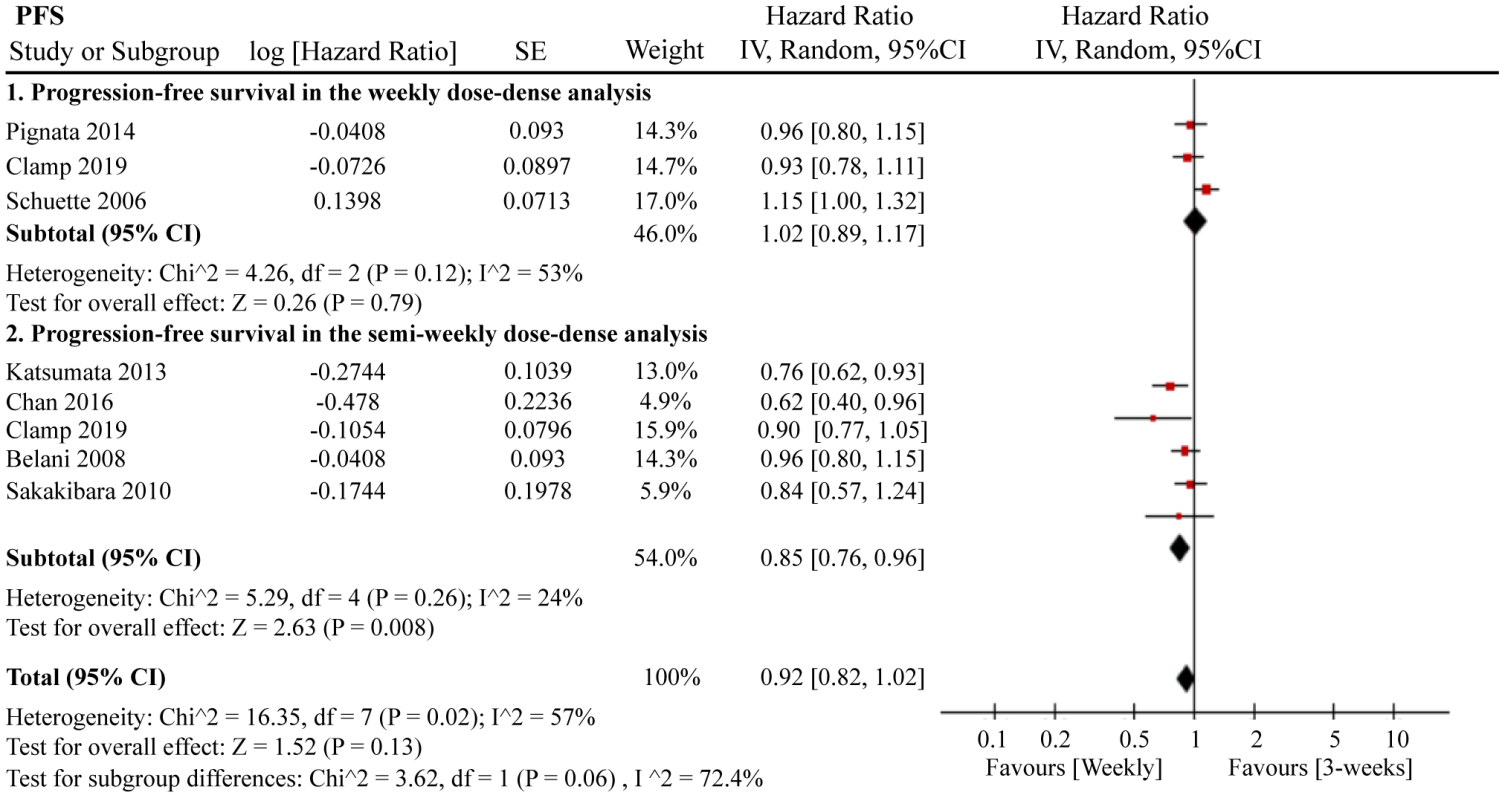
**The forest plot of HR for PFS in the subgroup analysis based on carboplatin administration schedules.** HR: hazard ratio; PFS: progression-free survival.

**Figure 4:**
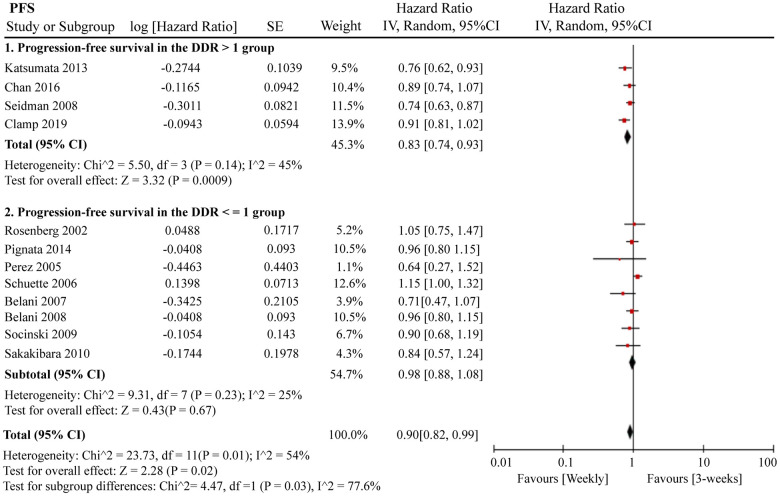
**The forest plot of HR for PFS in the subgroup analysis based on the DDR of paclitaxel.** HR: hazard ratio; PFS: progression-free survival; DDR: dose density ratio.

**Figure 5:**
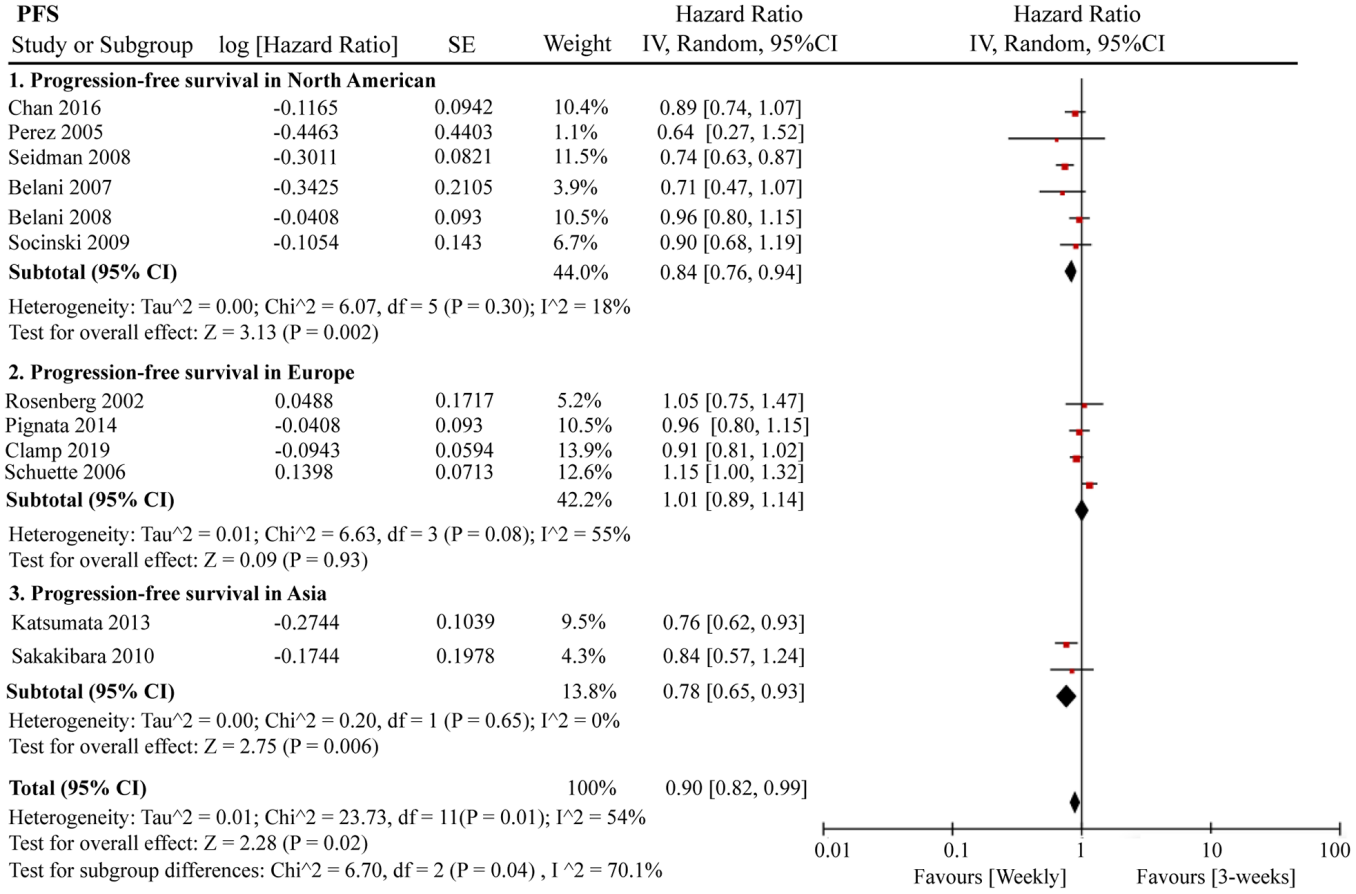
**The forest plot of HR for PFS in the subgroup analysis based on the ethnic differences.** HR: hazard ratio; PFS: progression-free survival.

A total of 12 RCTs were included for PFS analysis. In summary, the weekly paclitaxel regimen exhibited better PFS than the three-week paclitaxel regimen (HR = 0.90, 95%CI = 0.82–0.99, *P* = 0.02) (Figure 2). Other than paclitaxel, carboplatin was one of the main chemotherapy drugs used in the eligible RCTs under two administration schedules (area under the curve (AUC) = 2 or AUC = 5–6). According to Marchetti et al., carboplatin was defined as semi-weekly dose-dense (AUC = 5–6) and weekly dose-dense (AUC = 2) [33]. As shown in Figure 3, subgroup analysis revealed that semi-weekly dose-dense carboplatin favored better PFS than the three-week paclitaxel regimen (HR = 0.85, 95%CI = 0.76–0.96, *P* = 0.008), but weekly dose-dense carboplatin did not show the same benefit (HR = 1.02, 95%CI = 0.89–1.17, *P* = 0.79). We used the dose density ratio (DDR) to measure the significance of different doses of paclitaxel for each RCT with the formula DDR = [weekly (mg/m^2^/3–4wk)] / [Q3week (mg/m^2^/3wk)] according to Huang et al. [15]. Subgroup analysis revealed that the one-week paclitaxel regimen achieved better PFS than the three-week regime in the DDR >1 subgroup (HR = 0.83, 95%CI = 0.74–0.93, *P* = 0.0009). In the DDR < 1 subgroup, however, no significant difference in PFS was found between the two paclitaxel administration schedules (HR = 0.98, 95%CI = 0.88–1.08, *P* = 0.67) (Figure 4). Further subgroup analysis based on ethnic differences revealed that weekly paclitaxel regimen could improve patients’ PFS compared to 3-weeks paclitaxel regimen in North American (HR = 0.84, 95%CI = 0.76–0.94, *P =* 0.002) and Asia (HR = 0.78, 95%CI = 0.65–0.93, *P =* 0.006), but not in Europe (HR = 1.01, 95%CI = 0.89–1.14, *P =* 0.93) (Figure 5).

### One-week paclitaxel treatment did not obtain OS benefit compared to three-week paclitaxel treatment

In total, 13 eligible RCTs were included for OS analysis. In general, no significant difference in OS was found between the two paclitaxel regimens (HR = 0.98, 95%CI = 0.91–1.06, *P* = 0.62) (Supplementary Figure 1). Subgroup analyses were also performed. As shown in Supplementary Figures 2, 3, no significant differences in OS in the subgroups of the two paclitaxel schedules were detected: i.e., weekly dose-dense (HR = 1.07, 95%CI = 0.94–1.21, *P* = 0.32), semi-weekly dose-dense (HR = 0.92, 95%CI = 0.78–1.08, *P* = 0.31), DDR > 1 group (HR = 0.93, 95%CI = 0.73–1.17, *P* = 0.51), and DDR < 1 (HR = 1.05, 95%CI = 0.95–1.15, *P* = 0.33). There were no obvious differences between weekly and 3-weeks paclitaxel regimens regarding OS in North American (HR = 0.97, 95%CI = 0.81–1.15, *P* = 0.69), Europe (HR = 1.08, 95%CI = 0.95–1.21, *P* = 0.24), and Asia (HR = 0.88, 95%CI = 0.61–1.29, *P* = 0.52) (Supplementary Figure 4).

### Three-week paclitaxel favored better ORR

**Figure 6:**
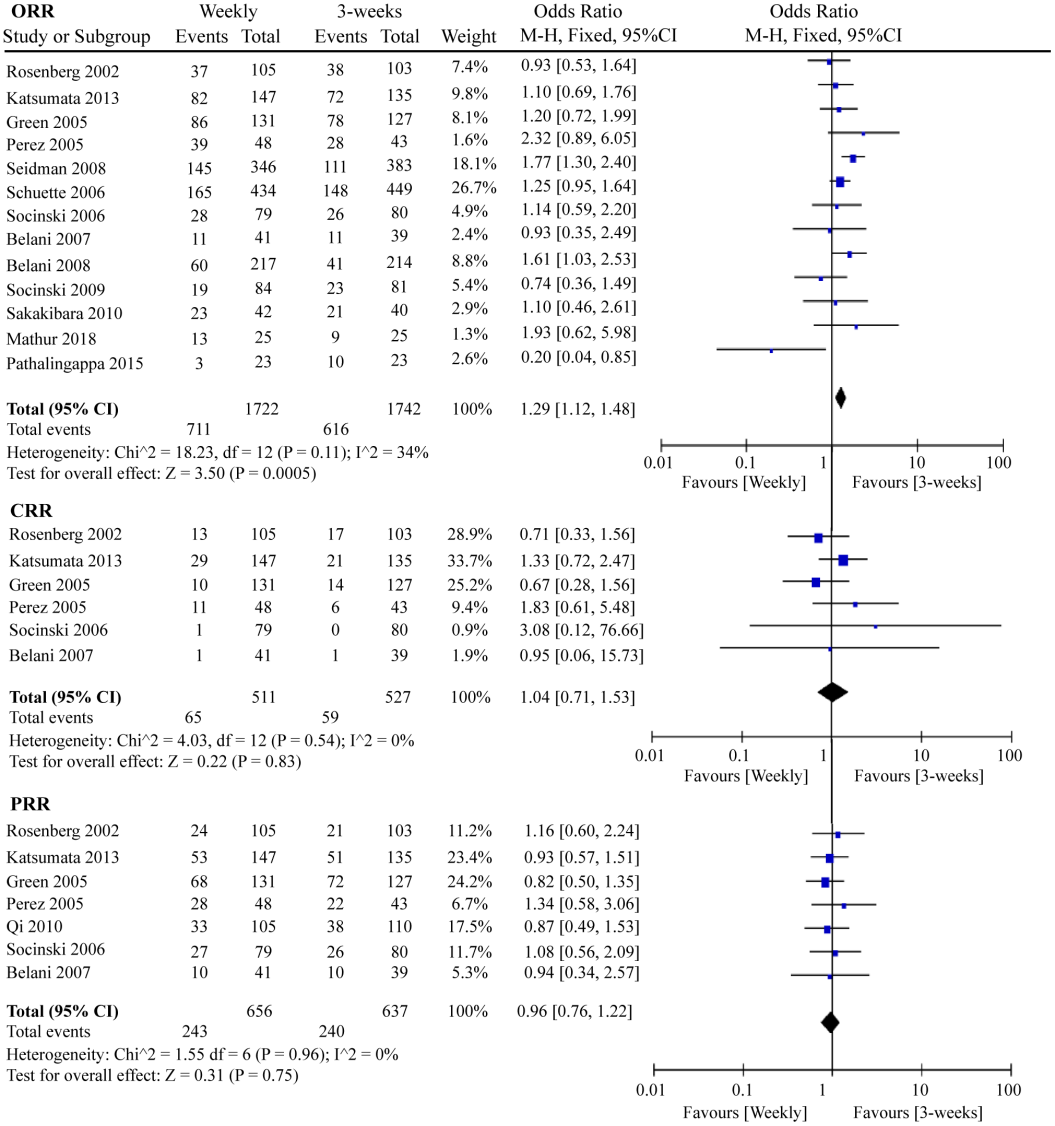
**The forest plot of OR for ORR, CRR, and PRR in the weekly paclitaxel compared to 3-weeks paclitaxel regimen, respectively.** OR: odds ratio; ORR: overall response rate; CRR: complete response rate; PRR: partial response rate.

Response rate is an important indicator for measuring the efficacy of chemotherapy drugs. In this meta-analysis, we determined the ORR, complete response rate (CRR), and partial response rate (PRR) of the two paclitaxel dosing regimens. As shown in Figure 6, 1 367 ORR events occurred in 13 RCTs containing 3 464 patients. Interestingly, the three-week paclitaxel regimen favored a better ORR than the weekly paclitaxel regimen (odds ratio (OR) = 1.29, 95%CI = 1.12–1.48, *P* = 0.0005). No obvious differences in CRR (OR = 1.04, 95%CI = 0.71–1.53, *P* = 0.83) or PRR (OR = 0.96, 95%CI = 0.76–1.22, *P* = 0.75) were observed between the one-week and three-week paclitaxel regimens.

### G3/4 adverse events in one-week and three-week paclitaxel treatment

**Figure 7:**
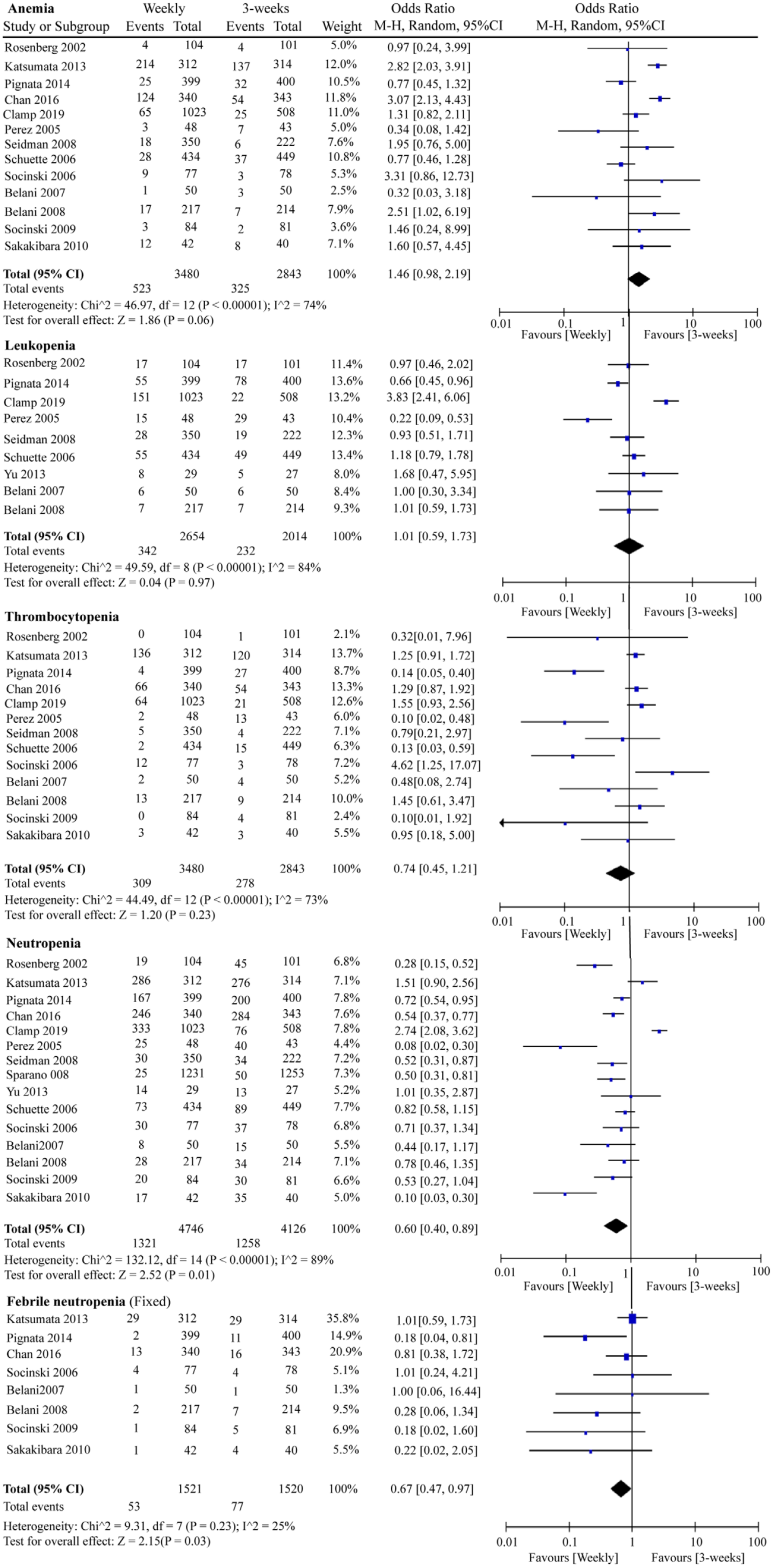
**The forest plot of OR for hematologic toxicities (anemia, leukopenia, thrombocytopenia, neutropenia, and febrile neutropenia) in the weekly paclitaxel compared to 3-weeks paclitaxel regimen.** OR: odds ratio.

We included G3/4 hematological and non-hematological toxicities in this RCT meta-analysis. Globally, serious adverse events were less likely to occur in the weekly paclitaxel regimen. As shown in Figure 7, chemotherapy-induced G3/4 neutropenia (OR = 0.60, 95%CI = 0.40–0.89, *P* = 0.01) and G3/4 febrile neutropenia (OR = 0.67, 95%CI = 0.47–0.97, *P* = 0.03) occurred less frequently under weekly paclitaxel treatment. Nevertheless, there were no significant differences in the incidences of G3/4 anemia (OR = 1.46, 95%CI = 0.98–2.19, *P* = 0.06), leukopenia (OR = 1.01, 95%CI = 0.59–1.73, *P* = 0.97), or thrombocytopenia (OR = 0.74, 95%CI = 0.45–1.21, *P* = 0.23) between the two different paclitaxel schedules. In terms of non-hematological adverse events, weekly paclitaxel showed a lower frequency of G3/4 arthritis (OR = 0.34, 95%CI = 0.17–0.66, *P* = 0.001) and G3/4 alopecia (OR = 0.31, 95%CI = 0.19–0.49, *P* < 0.00001) compared to the three-week paclitaxel regimen, but a higher occurrence of G3/4 diarrhea (OR = 1.65, 95%CI = 1.18–2.30, *P* = 0.003). No obvious differences in the occurrence of G3/4 vomiting (OR = 0.87, 95%CI = 0.61–1.23, *P* = 0.43), nausea (OR = 1.04, 95%CI = 0.77–1.39, *P* = 0.81), infection (OR = 1.11, 95%CI = 0.71–1.75, *P* = 0.64), fatigue (OR = 1.16 95%CI = 0.85–1.56, *P* = 0.35), dyspnea (OR = 1.14, 95%CI = 0.72–1.79, *P* = 0.57), constipation (OR = 0.78, 95%CI = 0.44–1.39, *P* = 0.40), or neuropathy (OR = 0.90, 95%CI = 0.54–1.50, *P* = 0.68) were detected between the two paclitaxel administration schedules (Supplementary Figure 5).

### Quality assessment and publication bias analysis of RCTs

**Figure 8:**
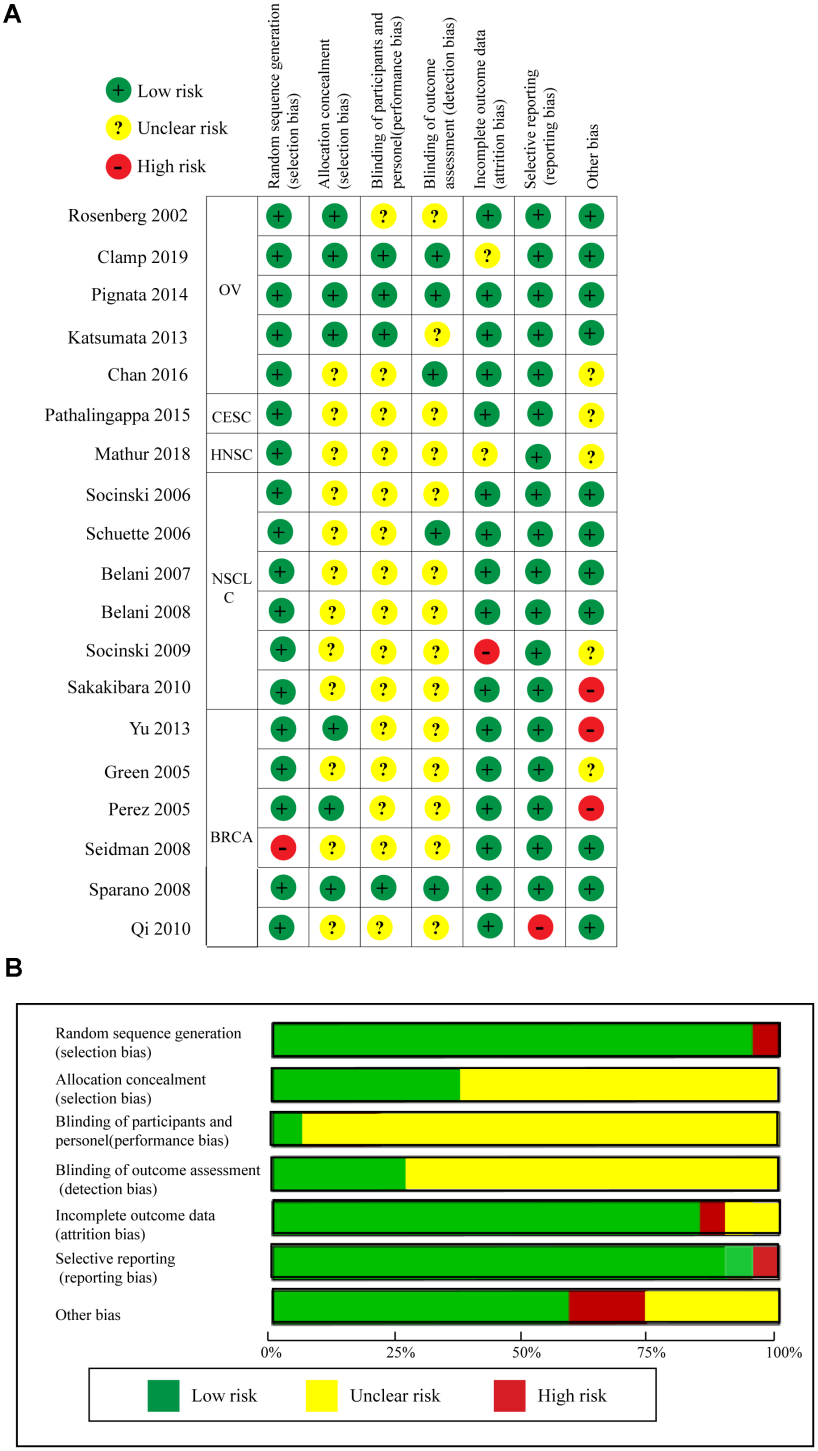
**Quality assessments of the included RCTs.** (**A**) Risk of bias summary. (**B**) Risk of bias graph.

Quality assessment of the 19 RCTs is shown in Figure 8 according to the Cochrane Collaboration risk of bias tool. The overall risk of the included RCTs was moderate. Undetermined risk was mainly due to a lack of relevant information. The funnel plot cannot really measure the publication bias of the included studies when the included studies were less than 10 [34]. Therefore, we only measured the publication bias of the included RCTs regarding OS, PFS, and ORR using the funnel plot. As shown in Supplementary Figure 6, publication biases regarding OS and ORR were not identified, but evident publication bias was found for PFS. Thus, we used the trim and fill method to correct for this bias [35, 36]. As seen in Supplementary Figure 7, blank1 (HR = 1.39, 95%CI = 0.59–3.29) and blank2 (HR = 1.00, 95%CI = 0.87–1.15) were used to correct the publication bias, and the adjusted pooled HR (HR = 0.93, 95%CI = 0.88–0.98, *P =* 0.004) for PFS still showed a significant difference.

## DISCUSSION

Paclitaxel is the cornerstone of first-line chemotherapy due to its significant anti-angiogenic effects. Both one-week and three-week paclitaxel administration are used as standard chemotherapy regimens for certain malignancies [16, 37, 38]. Weekly paclitaxel administration has a reliable theoretical basis and several preclinical models have proven that continuous low-dose paclitaxel administration has obvious anti-angiogenesis and pro-cell apoptosis effects [39]. In addition, several clinical trials have also indicated that a weekly paclitaxel regimen could provide more stable and greater dose intensity in the plasma, thereby enhancing paclitaxel exposure and intra-tumoral drug perfusion [40–43]. Therefore, it is difficult for tumor cells to recover quickly from paclitaxel-induced damage, resulting in a reduction of invasive or proliferative abilities [33, 44]. Here, we performed a systematic meta-analysis to compare the efficacies and toxicities of two paclitaxel administration schedules on pan-cancers. In total, 19 eligible RCTs containing 9 674 patients were included. Results revealed that, compared to the three-week paclitaxel administration schedule, weekly paclitaxel significantly prolonged PFS and demonstrated a lower incidence of serious adverse events in advanced malignant tumors.

Several included RCTs showed results consistent with our conclusions, confirming that weekly paclitaxel can significantly improve patients’ prognosis [7, 9, 25] and is better tolerated by patients [9, 19, 30]. For example, Katsumata et al. concluded that dose-dense weekly paclitaxel can significantly improve OS and PFS in advanced ovarian cancer patients [7]. Furthermore, Belani et al. indicated that weekly paclitaxel plus carboplatin or gemcitabine can decrease toxic events [9, 30]. However, Socinski et al. reported that ORR and survival outcomes are similar between the two paclitaxel administration schedules [31]. Clamp et al. also revealed that weekly paclitaxel regimen not only does not improve patient PFS, but also causes more G3/4 toxic events than that under the standard regimen [14]. In addition, other meta-analyses comparing the efficacy of paclitaxel administration schedules on ovarian, breast, and non-small cell lung cancers report inconsistent conclusions [33, 45, 46]. For example, Marchetti et al. concluded that the three-week paclitaxel regimen should remain as the standard therapy for ovarian cancer [33], whereas Mauri et al. suggested that weekly paclitaxel should be used for advanced/metastatic breast cancer due to OS advantages [46]. Based on these contradictions, we performed a meta-analysis to incorporate the latest high-quality RCTs to measure differences between the two paclitaxel regimens.

The doses of paclitaxel received by patients under the weekly or three-week paclitaxel regimens were not consistent. As such, DDR was used to balance the impact of paclitaxel dose differences on outcome evaluation. Results showed that weekly paclitaxel administration demonstrated better PFS than the three-week paclitaxel regime when DDR > 1. The carboplatin administration schedules also varied in the weekly paclitaxel regimen. Subgroup analysis revealed that the semi-weekly regimen (i.e., carboplatin (AUC = 5–6) + weekly paclitaxel) favored better PFS. Thus, we hypothesized that within a certain drug-dose range, there was a positive correlation between patient prognosis and paclitaxel/carboplatin dose. However, the appropriate concentration range for paclitaxel and carboplatin needs further study. Ethic differences are also important factors affecting the efficacy of paclitaxel. Studies have shown that the expression level of CYP450 enzyme varied greatly in different ethnic populations, which was reported to play major roles in paclitaxel metabolism [47, 48]. Here, we also found that weekly paclitaxel regimen could improve patients' PFS compared to 3-weeks paclitaxel regimen in North American and Asia, but not in Europe.

Our meta-analysis exhibits several specific strengths. While Huang et al. performed a similar meta-analysis on 10 RCTs in 2012 [15], only differences in G3 sensory neuropathy, G3/4 neutropenia, and response rate between one-week and three-week paclitaxel regimens were examined, and they mistakenly concluded that the weekly regimen favored a better response rate. Actually, we re-performed meta-analysis on these data, and concluded that 3-weeks regimen favored a better response rate (OR = 1.24, 95%CI = 1.01-1.52, *P =* 0.04) (Supplementary Figure 8). Here, we were the first meta-analysis to compare the differences of efficacy and toxicities between weekly and 3-weeks paclitaxel regimens on pan-carcinomas in last decade, which included 19 eligible RCTs and 9 674 patients. Here, we comprehensively analyzed the differences in prognosis (OS and PFS), response rate, and G3/4 toxicities between the two administration schedules. At the pan-cancer level, we found that weekly paclitaxel favored better PFS with a lower risk of G3/4 adverse events, whereas the three-week paclitaxel regimen favored better ORR, with CRR and PRR found to be similar between the groups. The higher ORR but poorer PFS in three-week regimen did not appear contradictory, which could be explained by differences in individual patient characteristics (e.g., age, sex), body status, drug resistance, and serious complications. Patient's tolerance and response rate to paclitaxel need to be fully considered at the same time. Although 3-weeks paclitaxel obtained a higher response rate, it was accompanied by a higher rate of adverse events. G3/4 neutropenia and febrile neutropenia are life-threatening toxicities, and G3/4 arthritis and alopecia also seriously affect the quality of patients' life. Actually, higher response rate to chemotherapy but no better prognosis has been proven in other cancers [49, 50].

This research should provide good guidance for clinicians and health policy makers. When selecting the most appropriate paclitaxel administration schedule, the efficiency, cost, toxicity, and treatment delays induced by adverse events should be seriously considered. The current meta-analysis revealed that the weekly paclitaxel regimen can significantly prolong PFS and decrease the incidence of adverse events. We realize the change in paclitaxel administration from three weeks to one week is a challenge for gynecological oncology due to the increase in the number of admissions and drug administration [21]. Cost-effectiveness is an important factor influencing treatment decisions. Chan et al. showed that the incremental cost-effectiveness ratio was $5 809 per progression-free life-year saved for weekly paclitaxel compared with three-week paclitaxel, and the weekly paclitaxel regimen was a cost-effective treatment option for advanced ovarian cancer using a Markov decision model [51]. Several studies have explored the economic benefits of paclitaxel in the treatment of advanced or metastatic breast cancer, but did not compare the differences between the two administration schedules [52, 53]. Prospective RCTs should clarify this in other malignant tumors in future studies.

Our meta-analysis has several limitations. Firstly, baseline characteristics of the enrolled patients varied among the RCTs, e.g., age, ECOG performance status, and clinical stage, which may affect outcome assessment. For example, elderly patients or those in poor physical condition tended to have poorer tolerance to chemotherapy drugs, thus leading to more adverse events and treatment delays. Secondly, a publication bias regarding PFS was found. Although this bias was corrected by the trim and fill method, the heterogeneity (I^^2^) of the included studies (RCTs plus two blanks) was 50%, which is the cut-off value. Therefore, we should be cautious when measuring PFS. Finally, some HRs for OS and PFS were not presented in the original manuscripts (Rosenberg 2002, Schuette 2006, and Perez 2005) [19, 26, 32], and were instead obtained from the survival curve.

In summary, compared to the three-week paclitaxel administration schedule, weekly paclitaxel treatment showed a PFS advantage and lower risk of complications. Thus, a weekly paclitaxel regimen is recommended as a first-line chemotherapy for advanced malignant tumors, especially for elderly patients in poor physical condition.

## MATERIALS AND METHODS

### Literature retrieval strategy

Our meta-analysis was carried out under the guidance of the PRISMA Statement [54]. To identify all relevant RCTs, we systematically searched four databases, including Cochrane Library, PubMed, Web of Science, and Clinical Trials.gov, from study inception to December 2020. The Medical Subject Heading (MESH) terms included “paclitaxel”, “weekly”, “dose-dense”, and “randomized”. Only RCTs were included in our study. Previous meta-analyses and systematic reviews were used for reference in detailed search strategies for each database [15, 33, 45, 46, 55]. Details on the literature retrieval strategy are described in Supplementary Table 1. We have registered this meta-analysis in PROSPERO, and the granted number is CRD42020213815.

### Eligible study selection

Those RCTs that compared the efficacy and/or adverse events of one-week and/or three-week paclitaxel regimens were considered eligible. In addition to paclitaxel, carboplatin and other adjuvant chemotherapy drugs were also included in the two different paclitaxel administration groups. RCTs were excluded if they were not written in English, showed a small sample size (n < 10), or showed no measurable outcomes.

### Data extraction and quality assessment

We focused on the reported OS, PFS, ORR, CRR, PRR, and G3/4 adverse events. Details on the included studies were extracted, including first author’s name, country, starting time of trial, number of patients, patient characteristics, and interventions (detailed drug administration schedules). We performed quality assessment of the included RCTs according to the Cochrane Collaboration risk of bias tool [56]. Seven bias risks were included: i.e., random sequence generation, allocation concealment, blinding of participants and personnel, blinding of outcome assessment, incomplete outcome data, selective reporting, and others. The relevant research work was performed independently by two reviewers (Ting Peng and Shitong Lin). Divergences in opinion were resolved through discussion and consensus between the two reviewers or by discussion with Professor Cai Cheng.

### Outcome and subgroup analysis

Data outcomes were extracted from the published articles with the longest follow-up. Primary outcomes were OS and PFS. Secondary outcomes included ORR, PRR, CRR, and G3/4 hematological and non-hematological toxicities.

We performed subgroup analysis to further measure the effects of different paclitaxel administration schedules. Firstly, the doses of paclitaxel received by patients in each treatment cycle for the two different administration schedules were not the same. We used the DDR to measure the effects of dose differences on outcome assessment. Here, DDR ([weekly (mg/m^2^) × 3/3–4wk)] / [3 weeks (mg/m^2^)/3–4wk)]) refers to the ratio of the weekly paclitaxel dose received by patients under the two paclitaxel administration regimens over each course of treatment [15]. Secondly, paclitaxel plus carboplatin was the main chemotherapy regimen, and carboplatin was used under two different administration schedules (AUC = 2 or AUC = 5–6) [33]. Thus, we used the DDR and carboplatin administration schedules to perform subgroup analysis. Finally, the populations of included studies were mainly from North American, Europe, and Asian countries, and we also performed subgroup analysis according to their different races.

### Statistical analysis

RevMan v5.3 software was used for statistical analyses. The hazard ratio (HR), 95% CI, standard error (SE), and log [HR] for OS and PFS were extracted or calculated from published RCT data according to the methods described by Tierney et al. [57]. The ORR, CRR PRR, and G3/4 toxicity events in eligible RCTs were extracted from published studies. For those RCTs in which survival HR was not revealed in the report, it was estimated from the provided survival-curve using the Engauge Digitizer. The 95%CI of the survival HR was estimated using the delta method [57]. The small square and its size represents the HR (or OR) and weight of each RCT in the forest, respectively. The horizontal line through the small square represents the 95%CI. The small diamond at the bottom of the forest plot represents the pooled HR (or OR), and its width represents the 95%CI. Cochran’s Q test and I^2^ statistics were used to evaluate the heterogeneity of the included RCTs. Statistical heterogeneity was considered when *P* < 0.10 or I^2^ > 50%, with the random-effects model used in this case; otherwise, the fixed-effects model was applied. A funnel plot was used to show potential publication bias when there were enough eligible articles. *P* < 0.05 was defined as a significant two-way *P*-value. All measured endpoints were presented in the form of forest maps.

### Data availability

As the secondary user of deidentified patient-level clinical trial data, we are not authorized to share the data based on the data use agreements.

## Supplementary Material

Supplementary Figures

Supplementary Table 1
