# Supplementary material for: Comparison of one-week versus three-week paclitaxel for advanced pan-carcinomas: systematic review and meta-analysis
Source: Aging (Albany NY). 2022 Feb 26;14(4):1959–82. doi: 10.18632/aging.203919 (PMC8908930; doi:10.18632/aging.203919)
Supplement: Supplementary Figures [file aging-14-203919-s001.pdf]

SUPPLEMENTARY FIGURES

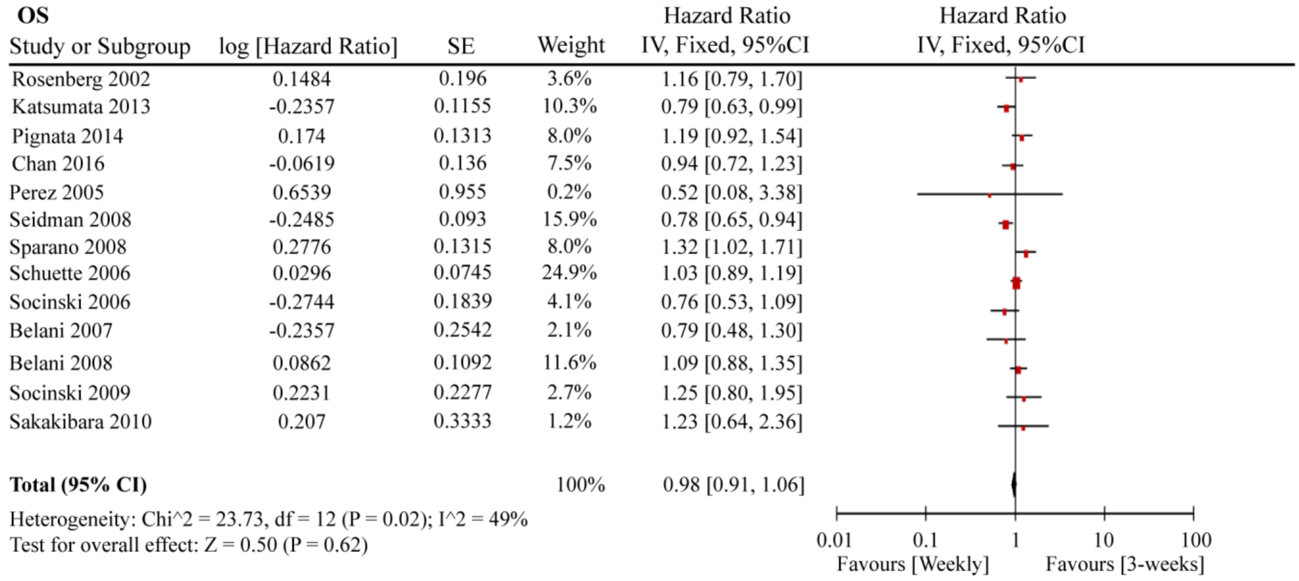

Supplementary Figure 1. The forest plot of HR for OS in the weekly paclitaxel compared to 3-weeks paclitaxel regimen. HR: hazard ratio; OS: overall survival.

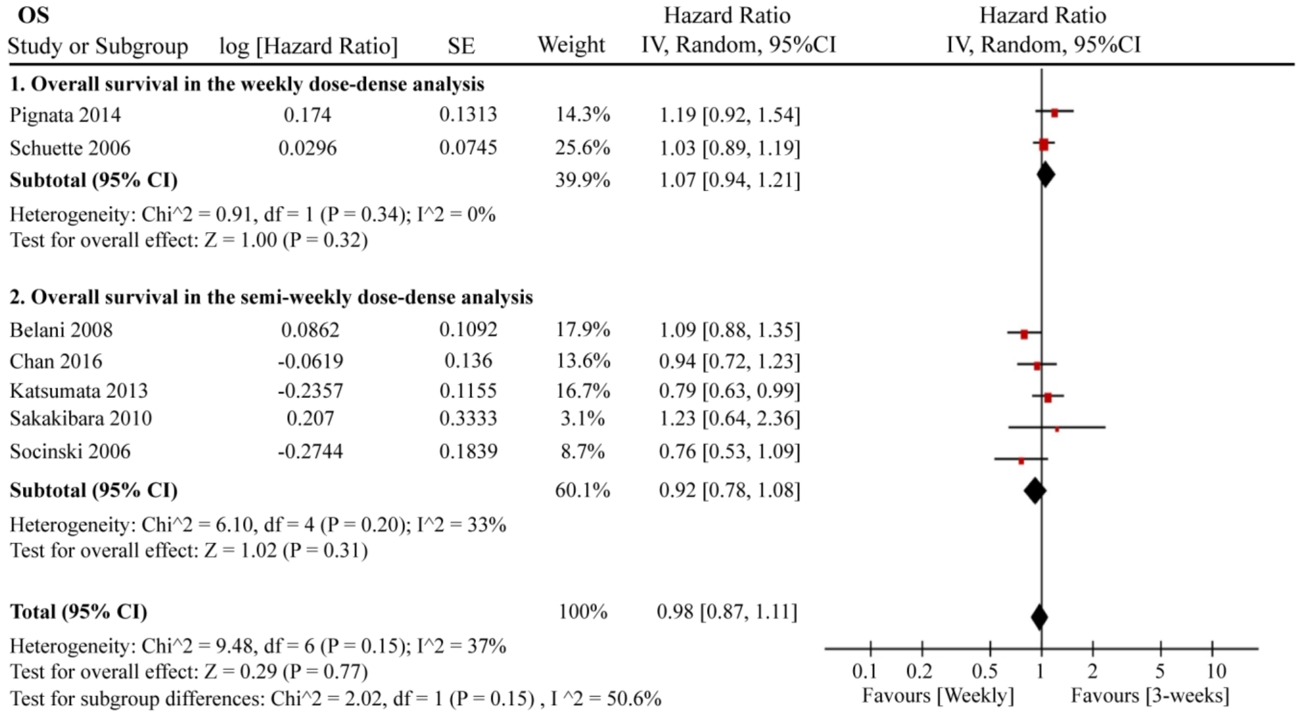

Supplementary Figure 2. The forest plot of HR for OS in the subgroup analysis based on carboplatin administration schedules. HR: hazard ratio; OS: overall survival.

OS

Study or Subgroup	log [Hazard Ratio]	SE	Weight	Hazard Ratio IV, Random, 95%CI
**1. Overall survival in the DDR > 1 group**				
Katsumata 2013	-0.2357	0.1155	10.6%	0.79 [0.63, 0.99]
Chan 2016	-0.0619	0.136	9.1%	0.94 [0.72, 1.23]
Seidman 2008	-0.2485	0.093	12.4%	0.78 [0.65, 0.94]
Sparano 2008	0.2776	0.1315	9.4%	1.32 [1.02, 1.71]
**Tubtotal (95% CI)**			41.4%	0.93 [0.73, 1.17]
Heterogeneity: Chi^2 = 12.21, df = 3 (P = 0.007); I^2 = 75%				
Test for overall effect: Z = 0.65 (P = 0.51)				
**2. Overall survival in the DDR < = 1 group**				
Rosenberg 2002	0.1484	0.196	5.9%	1.16 [0.79, 1.70]
Pignata 2014	0.174	0.1313	9.4%	1.19 [0.92 1.54]
Perez 2005	-0.6539	0.955	0.4%	0.52 [0.08, 3.38]
Schuette 2006	0.0296	0.0745	14.0%	1.03 [0.89, 1.19]
Socinski 2006	-0.2744	0.1839	6.4%	0.76 [0.53, 1.09]
Belani 2007	-0.2357	0.2542	4.1%	0.79 [0.48, 1.30]
Belani 2008	0.0862	0.1092	11.0%	1.09 [0.88, 1.35]
Socinski 2009	0.2231	0.2277	4.8%	1.25 [0.80, 1.95]
Sakakibara 2010	0.207	0.3333	2.6%	1.23 [0.64, 2.36]
**Subtotal (95% CI)**			58.6%	1.05 [0.95, 1.15]
Heterogeneity: Chi^2 = 7.04, df = 8 (P = 0.53); I^2 = 0%				
Test for overall effect: Z = 0.98 (P = 0.33)				
**Total (95% CI)**			100.0%	0.99 [0.88, 1.11]
Heterogeneity: Chi^2 = 23.73, df = 12 (P = 0.02); I^2 = 49%				
Test for overall effect: Z = 0.17 (P = 0.87)				
Test for subgroup differences: Chi^2 = 0.95, df = 1 (P = 0.33), I^2 = 0%				

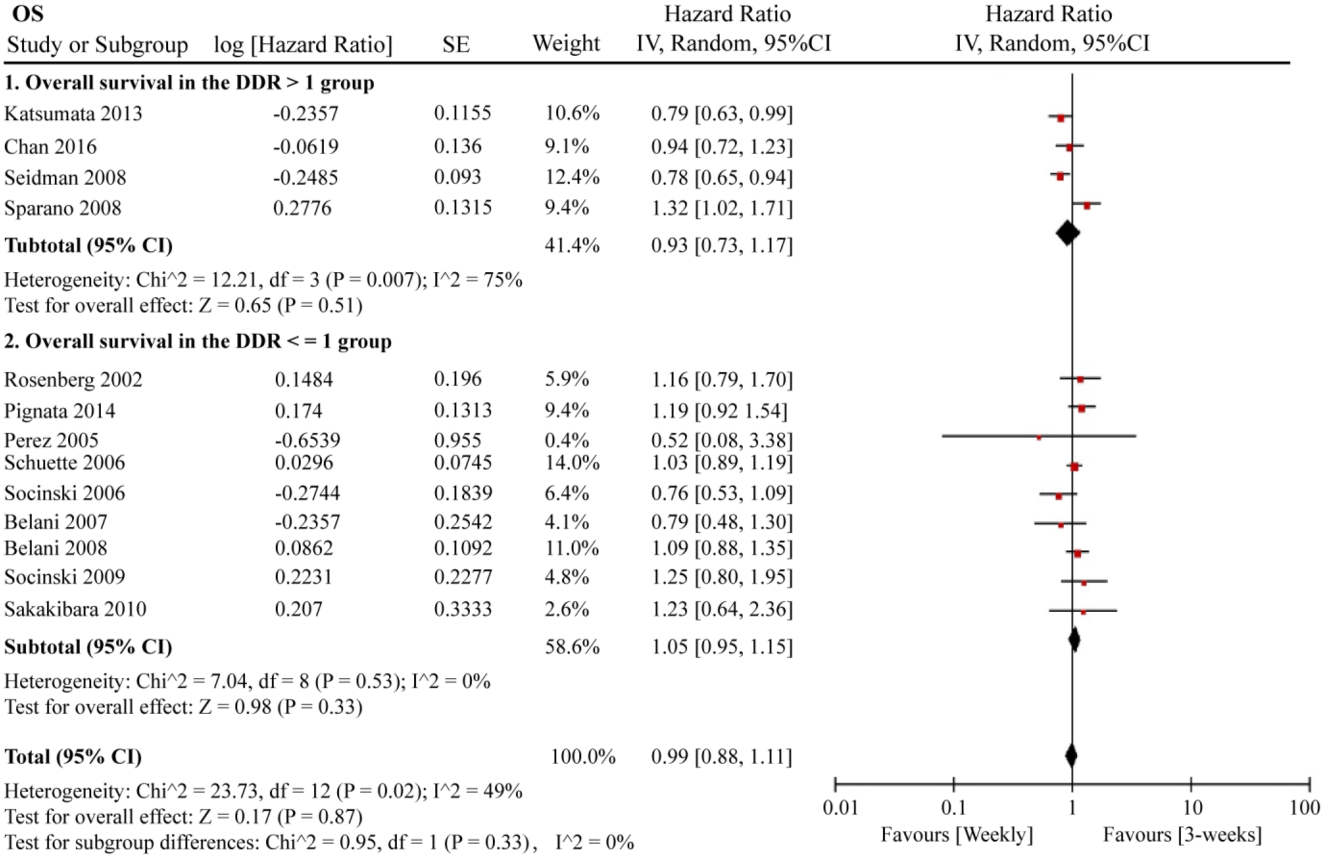

Supplementary Figure 3. The forest plot of HR for OS in the subgroup analysis based on the DDR of paclitaxel. HR: hazard ratio; OS: overall survival; DDR: dose density ratio.

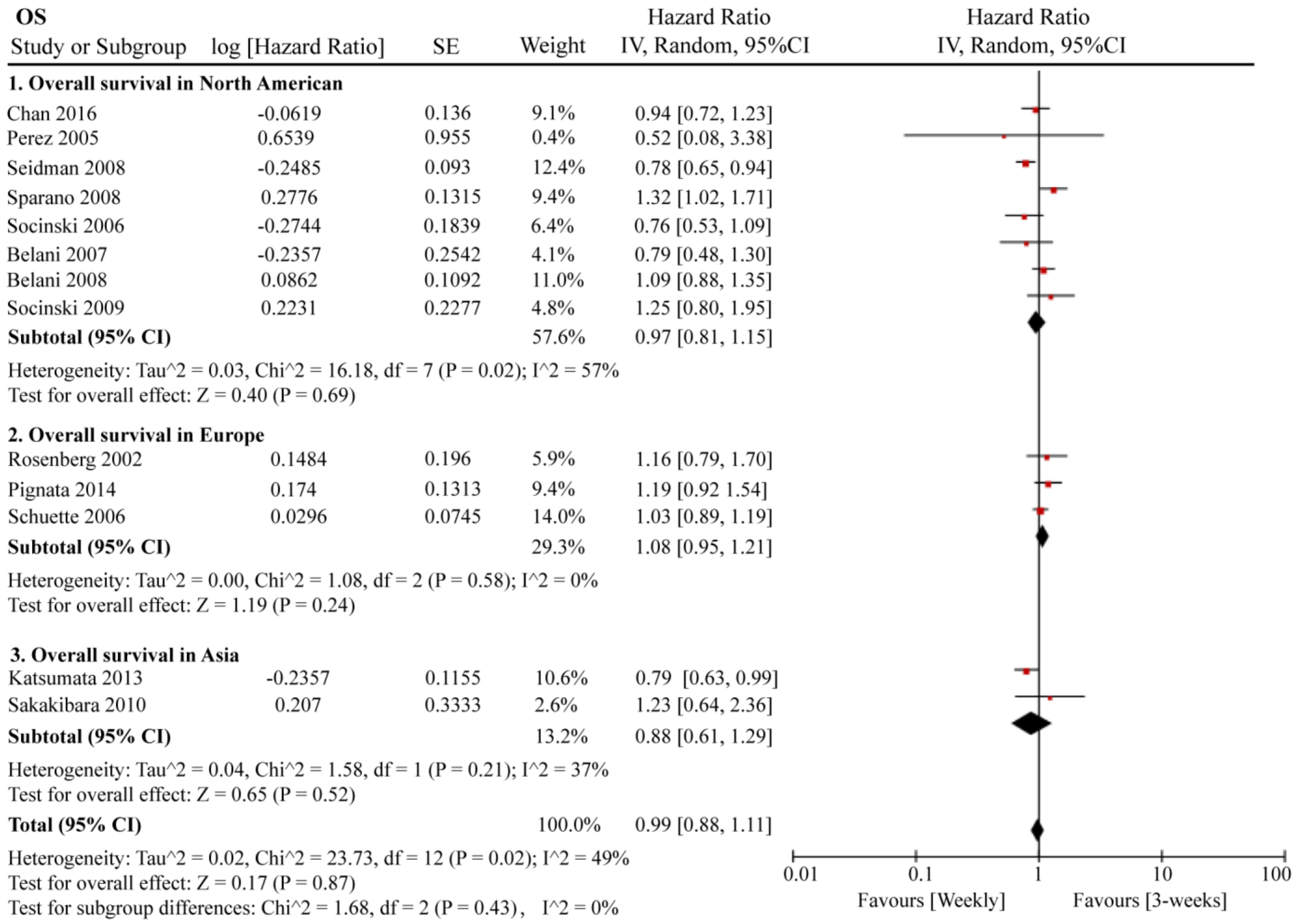

Supplementary Figure 4. The forest plot of HR for OS in the subgroup analysis based on ethnic differences of included populations. HR: hazard ratio; OS: overall survival.

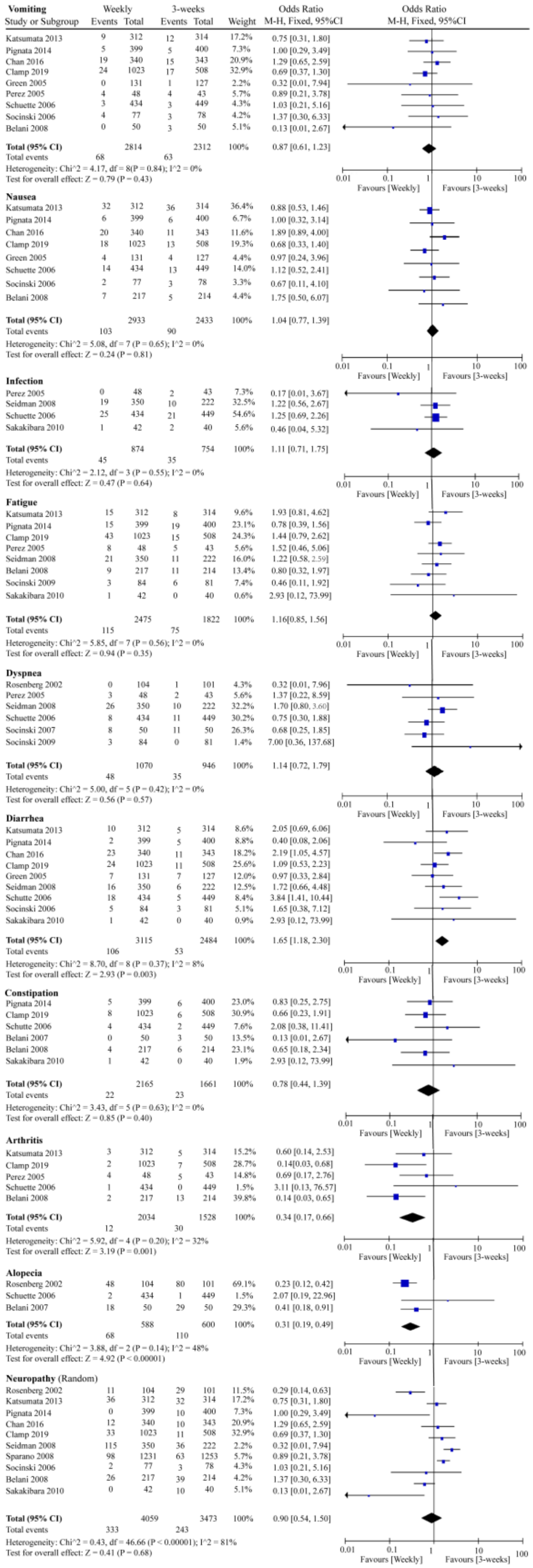

Supplementary Figure 5. The forest plot of OR for non-hematologic toxicities (vomiting, nausea, infection, fatigue, dyspnea, diarrhea, constipation, arthritis, alopecia, and neuropathy) in the weekly paclitaxel compared to 3-weeks paclitaxel regimen. OR: odds ratio.

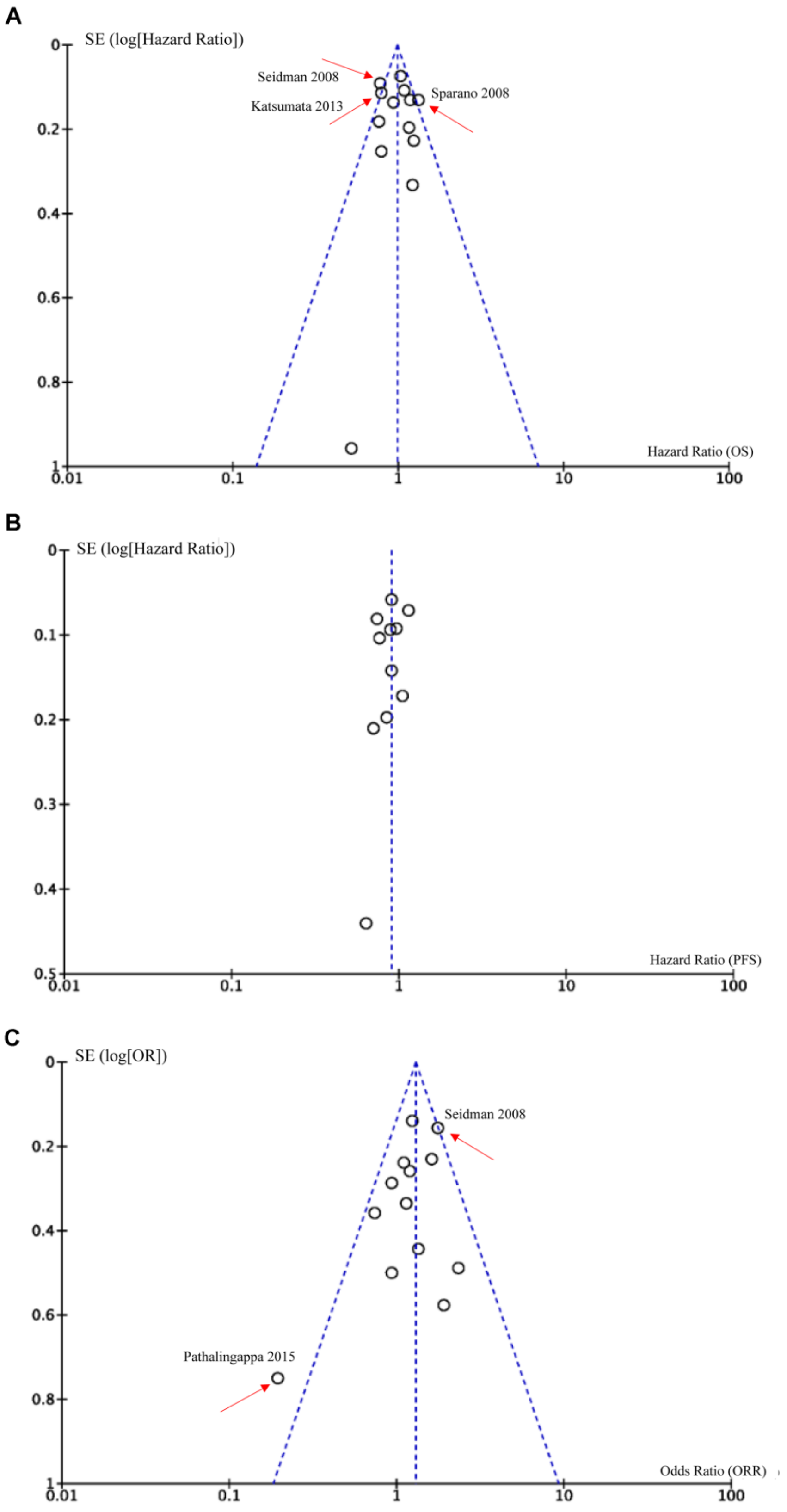

Supplementary Figure 6. The funnel plots for publication bias. (**A**) for articles measuring the incidence of OS. (**B**) for articles measuring the incidence of PFS. (**C**) for articles measuring the incidence of ORR. The vertical blue dotted line and two oblique blue dotted lines represent the position of the combined effect value and its corresponding 95% confidence interval on the x-axis, respectively. The red arrows point to outlier studies. OS: overall survival; PFS: progression-free survival; ORR: overall response rate.

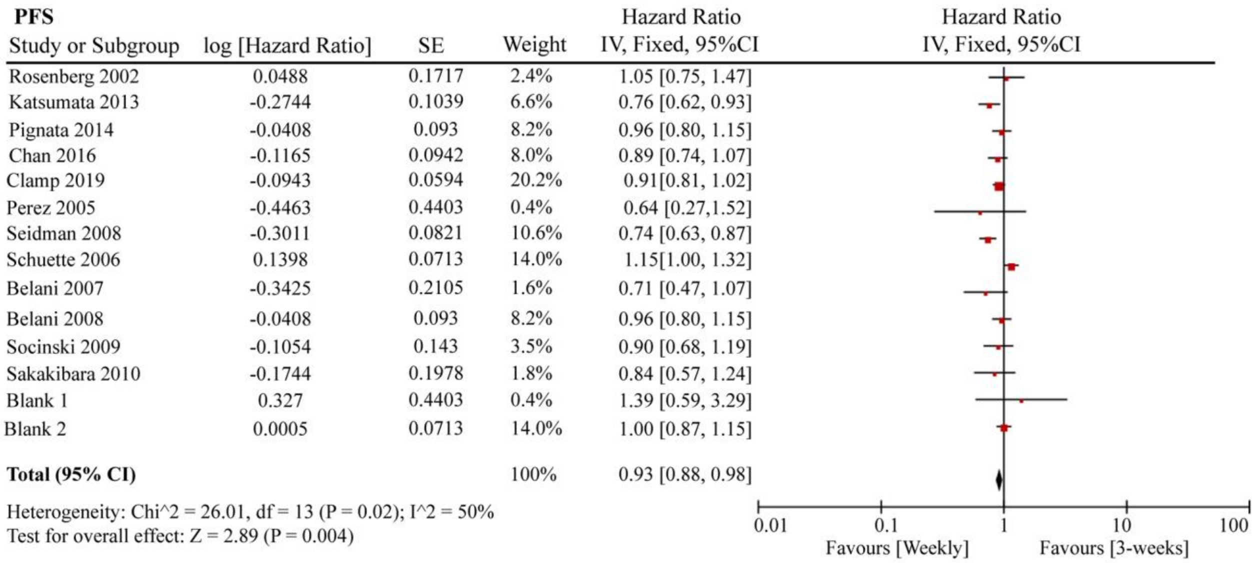

Supplementary Figure 7. The forest plot of adjusted HR by trim and fill for PFS in the weekly paclitaxel compared to 3-weeks paclitaxel regimen. HR: hazard ratio; PFS: progression-free survival.

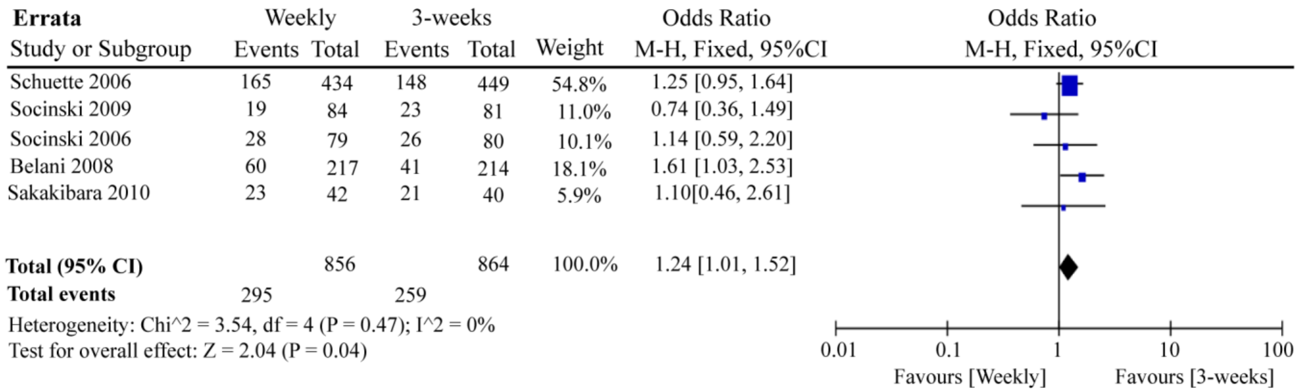

Supplementary Figure 8. The corrected forest plot of OR for response rate in the weekly paclitaxel compared to 3-weeks paclitaxel regimen.
