# Supplementary material for: Comparison of one-week versus three-week paclitaxel for advanced pan-carcinomas: systematic review and meta-analysis
Source: Aging (Albany NY). 2022 Feb 26;14(4):1959–82. doi: 10.18632/aging.203919 (PMC8908930; doi:10.18632/aging.203919)
Supplement: Supplementary Table 1 [file aging-14-203919-s002.pdf]

SUPPLEMENTARY TABLE

Supplementary Table 1. The search strategies for related articles in four included databases.

PubMed:
1. Drug Therapy [Mesh]
2. Paclitaxel [Title/Abstract]
3. 1 OR 2
4. dose-dense [Title/Abstract]
5. weekly [Title/Abstract]
6. 4 OR 5
7. randomized controlled trial [Publication Type]
8. Randomized [Title/Abstract]
9. 7 OR 8
10. 3 AND 6 AND 9

Web of Science:
1. TI= paclitaxel
2. TI= (dose-dense OR weekly)
3. TS= (randomized controlled trial OR randomized OR placebo)
4. 1 AND 2 AND 3

Cochrane Library:
1. Neoplasms [Mesh]
2. neoplas* OR cancer* OR carcinom* OR malignan* OR tumor* OR tumour* [Title/Abstract]
3. 1 OR 2
4. weekly [Title/Abstract]
5. dose-dense [Title/Abstract]
6. 4 OR 5
7. Randomized controlled trial [Publication Type]
8. Randomized [Title/Abstract]
9. 7 OR 8
10. Paclitaxel [Title/Abstract]
11. 3 AND 6 AND 9 AND 10

ClinicalTrials.gov:
(Paclitaxel) AND (dose-dense OR weekly)
